# Star Shaped Long Chain Branched Poly (lactic acid) Prepared by Melt Transesterification with Trimethylolpropane Triacrylate and Nano-ZnO

**DOI:** 10.3390/polym10070796

**Published:** 2018-07-19

**Authors:** Le Yang, Zaijun Yang, Feng Zhang, Lijin Xie, Zhu Luo, Qiang Zheng

**Affiliations:** 1 Department of Polymer Materials and Engineering, College of Materials and Metallurgy, Guizhou University, Guiyang 550025, China; gs.leiyang15@gzu.edu.cn (L.Y.); gs.zjyang15@gzu.edu.cn (Z.Y.); gs.fengz16@gzu.edu.cn (F.Z.); gs.ljxie16@gzu.edu.cn (L.X.); 2 Department of Polymer Science and Engineering, Zhejiang University, Hangzhou 310027, China

**Keywords:** Poly (lactic acid), long chain branched, transesterification, star-shaped, melt strength

## Abstract

Long chain branched poly (lactic acid) (LCBPLA) was prepared via transesterification between high molecular weight poly (lactic acid) (PLA) and low molar mass monomer trimethylolpropane triacrylate (TMPTA) during melt blending in the presence of zinc oxide nanoparticles (nano-ZnO) as a transesterification accelerant in a torque rheometer. Compared with the traditional processing methods, this novel way is high-efficiency, environmentally friendly, and gel-free. The results revealed that chain restructuring reactions occurred and TMPTA was grafted onto the PLA backbone. The topological structures of LCBPLA were verified and investigated in detail. It was found that the concentration of the accelerants and the sampling occasion had very important roles in the occurrence of branching structures. When the nano-ZnO dosage was 0.4 phr and PLA was sampled at the time corresponding to the reaction peak in the torque curve, PLA exhibited a star-shaped topological structure with a high branching degree which could obviously affect the melt strength, extrusion foaming performances, and crystallization behaviors. Compared with pristine PLA, LCBPLA showed a higher melt strength, smaller cell diameter, and slower crystallization speed owing to the synergistic effects of nano-ZnO and the long chain branches introduced by the transesterification reaction in the system. However, severe degradation of the LCBPLAs would take place under a mixing time that was too long and lots of short linear chains generated due to the excessive transesterification reaction, with a sharp decline in melt strength.

## 1. Introduction

At present, the sustainable utilization of resources is arousing humans’ attention more and more. Poly (lactic acid) (PLA) is recognized as one of the most potential renewable resource-based biodegradable polymers due to its excellent performances such as high-mechanical properties, ease of process, good biocompatibility, and environmental-friendly characteristics [1,2,3,4,5,6]. Thanks to these advantages, PLA possesses numerous applications like being used as packaging materials, biomedical materials, and for films and containers. However, due to its linear structure and poor melt strength [7,8,9,10], it is a big challenge for PLA to be applied into some processes where entanglement properties and melt strength are dominant, such as thermoforming, continuous extrusion foaming, and film blowing. Therefore, the modification of PLA to enhance its melt strength is imperative for some specific applications.

The introduction of a long chain branched (LCB) structure into a polymer to enhance its matrix melt strength has been proved to be an efficient method. A fraction of LCB structures in PLA are able to enhance the melt strength remarkably owing to the augment of the entanglements among the macromolecule chains, and LCB macromolecules which possess an excellent melt strength and strain-hardening ability have already been confirmed in LCB polypropylene (LCBPP) [11,12] and LCBPLA [13]. In addition, crystallization behavior can be altered owing to the introduction of branches for branching PLA [14].

Generally, LCBPLA can be prepared via in situ polymerization in a reactor or by the method of melt reactive processing [15,16,17,18,19,20]. As a simple and effective method, melt reactive processing is mostly applied owing to its unique advantages such as its solvent-free process, short reactive time, low cost, and energy saving property. Presently, melt reactive processing for LCBPLA preparation mainly includes two methods: one is reactive processing via a melt chain extension reaction with an end group using multifunctional monomers as the branching center and the other is a radical reaction initiated by a peroxide initiator or high energy ray. Liu et al. [15,16] prepared LCBPLA via melt chain extension by the successive reactions of the end hydroxyl groups (-OH) of PLA with polyfunctional monomers. It was found that the star-like or tree-like LCB structure generated in the reactions contributed remarkably to the enhancement of strain-hardening under elongational flow, which improved the foaming ability substantially. Cailloux et al. [17,18] prepared LCBPLA sheets by a one-step reactive extrusion calendering process using a styrene-acrylic multi-functional-epoxide oligomeric agent, Joncryl-ADR-4300F, as the branching agent. It was found that tree arm star PLA can be obtained, which possessed a high melt elasticity and long relaxation time. You et al. [19] prepared LCBPLA by the means of a radical reaction in a molten state in the presence of a peroxide initiator and multifunctional monomer. The results indicated that the storage modulus (*G’*) and viscosity (η*) increased when PLA was long-chain branched and the crystallization rate parameter was improved sharply. Li et al. [20] investigated the extrusion foaming performance of LCBPLA prepared by UV-induced reaction extrusion with trimethylolpropane triacrylate (TMPTA). It was found that the advantage of LCBPLA on foaming was remarkable at a high temperature and high pressure, which would benefit the bead foaming and continuous extrusion foaming of PLA. However, disadvantages for these two methods are apparent. A drawback of end group reactions is their slow reaction kinetics, that is, the branching reaction may take more than 30 min to complete, while the residence time in typical melt processing equipment is less than 5 min [21]. Additionally, crosslinking and gel are difficult to avoid for a radical reaction initiated by peroxide or high energy ray.

Transesterification widely occurs in the processing of ester polymer melting mixing. The transesterification reaction concerning PLA blends has been investigated by many authors, and the representatives are the blends of PLA and poly(ε-caprolactone) (PCL) [22,23,24], blends of PLA and poly(3-hydroxybutyrate-co-3-hydroxyhexanoate) (PHBHHX) [25], and blends of PLA and poly(butylene adipate-co-terephthalate) (PBAT) [26,27,28,29,30], which have indeed developed a new technology for the synthesis of block or random copolyesters. However, the transesterification reaction between small molecular ester monomers and PLA macromolecules in blending processing, despite their interesting perspectives, was not much investigated. Slivniak et al. [31,32,33] synthesized multiblock copolyesters based on purified ricinoleic (RA) and lactic acids (LA) by the transesterification of high molecular weight PLA with ricinoleic acid and repolyesterification. Géraldine et al. [34] prepared some kind of low molecular weight PLA telechelic oligomers end-capped with acrylate groups by a transesterification reaction which was carried out in solution with a Lewis acid titanium catalyst using a high molecular weight PLA and a low molar mass diacrylate. Despite all this, transesterification reactions between small molecular ester monomers and PLA macromolecules in the melt state, as well as the applications, have been scarcely reported so far. The formation of PLA star branched macromolecules by transesterification with a small molecular multifunctional monomer has not been reported, although the efficiency of transesterification is higher than that of an extension reaction. It is one of the major reasons that the process conditions of the transesterification reaction between small molecules and macromolecular chains in a molten state is difficult to control and apply. An appropriate reaction time is the necessary requirement for LCBPLA, which is prepared by a melt transesterification reaction. Perfect LCBPLA cannot be formed by inadequate reactions under an insufficient mixing time, while a reaction that is too long has the same consequence owing to the degradation of LCBs caused by the excessive transesterification reaction.

In this work, the star shaped LCBPLA was prepared via the transesterification between high molecular weight PLA and small molecule TMPTA during melt blending. Compared with the traditional processing methods, this novel method is high-efficiency, environmentally friendly, and gel-free. The excellent catalytic properties and adsorption capacities of some nanometer materials have received increasing attention in recent years owing to their high surface energy and large specific surface area [35]. The environmentally friendly ZnO nanoparticles were applied as the efficient accelerant to enhance the transesterification reactivity of PLA with a multifunctional monomer to prepare a kind of star-shaped long chain branched poly (lactic acid) during melt blending. Additionally, the TMPTA was used as the branching centre. The structure and rheological properties of these blending PLA samples with different reaction times corresponding to the characteristics of the torque curve, as well as their topological structures and crystallization behaviors, were investigated in this paper. Moreover, the effect of the ZnO nanoparticles on the transesterification was also discussed in our study. 

## 2. Experimental

### 2.1. Materials

The PLA pellets (2003D, with 4.1% D enantiomer) used in this study were purchased from NatureWorks LLC Co. (Blair, NE, USA), and had a density of 1.24 g·cm^−3^, MFR (190 °C and 2.16 kg) of 4.86 g/10 min, *M*_w_ of 185293 g·mol^−1^, and PDI of 1.70. TMPTA was acquired from Jiangsu Hengyang Chemical Product Sales Department (Liyang, China). ZnO nanoparticles (99.8% metals basis, 50 ± 10 nm, surface treated by stearic acid) were obtained from Macklin Chemical Reagent LLC Co. (Shanghai, China). PLA pellets were dried at 60 °C for 24 h under vacuum before mixing.

### 2.2. Sample Preparation and Reactive Processing

**Figure 1:**
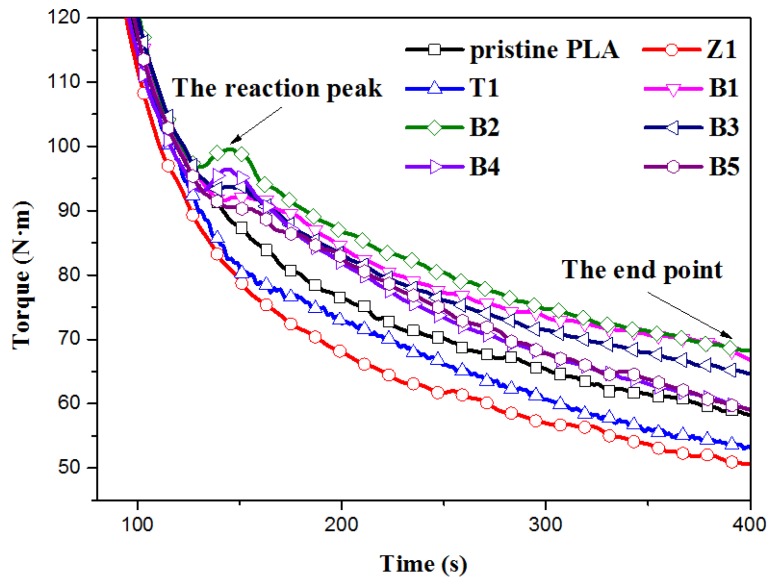
Torque curves of PLA samples at 190 °C.

In the premixing process, TMPTA was firstly dissolved in a mass of alcohol and ZnO nanoparticles were fully dispersed in this solution under ultrasonic conditions for 2 h. Then, the suspension liquid was sprayed evenly in the PLA pellets, and dried for 4 h in an oven at 40 °C to volatilize alcohol. Finally, the mixtures were stirred again for 2 min to ensure that they were mixed sufficiently before melt blending. Then, the modification of PLA (350 g) was carried out with a rotation speed of 80 rpm in a torque rheometer (three heating zones, 5 L in volume, Shanghai, China) at 190 °C for a preset time (corresponding to the reaction peak and the end point shown in Figure 1). Finally, the modified PLA samples were smashed to granules in a pulveriser. The detailed processing conditions and the formulations are listed in Table 1, and the corresponding torque curves are shown in Figure 1.

### 2.3. Purification of Sample

The reacted PLA samples were dissolved in dichloromethane, and then the solutions were charged into methyl alcohol at room temperature. The PLA sample was precipitated out, while the unreacted monomer remained soluble. The sediments were separated by filtration, ultrasonically absterged with methyl alcohol three times, and then were dried at 80 °C under vacuum for 24 h. The dried purified sample was used for the subsequent characterization.

### 2.4. Gel Determination

The smashed samples were packed with a phosphor bronze net of 400 meshes and extracted in boiling dichloromethane for 24 h in a Soxhlet extractor. In fact, no insoluble substance was found for all the samples in our study, implying that no gel was formed.

### 2.5. ^1^H Nuclear Magnetic Resonance Spectroscopy

Nuclear magnetic resonance (NMR), JEOL-ECX500 (JEOL, Tokyo, Japan), was used to characterize pristine PLA and modified PLA samples. The purified samples were dissolved in deuterated chloroform (CDCl_3_) at room temperature for ^1^H-NMR measurements.

### 2.6. X-ray Photoelectron Spectroscopy

X-ray photoelectron spectroscopy (XPS, K-Alpha^+^, ThermoFisher Scientific, Waltham, MA, USA) was applied to characterize the functional groups and the coordination effect of nano-ZnO in PLA. Sample B2 was dissolved in dichloromethane for 24 h and the nano-ZnO was extracted by centrifugation with a speed of 8000 rpm for 5 min. The extractive was obtained by filtration, ultrasonic washing five times with dichloromethane, and drying under vacuum at 80 °C for the measurements. Survey XPS spectra were acquired with a constant pass energy of 100 eV, while the high-resolution XPS spectra were recorded with a constant pass energy of 30 eV with a step length of 1.0 eV.

### 2.7. Fourier Transform Infrared Spectroscopy (FTIR)

In situ infrared spectroscopy and attenuated total reflectance (ATR) infrared spectroscopy were performed using a Fourier transform infrared spectrometer (Nicolet iS50, ThermoFisher Scientific, Waltham, MA, USA). Dried PLA pellets and TMPTA were first dissolved in dichloromethane and then ZnO nanoparticles were uniformly ultrasound dispersed in this solution. Volatilization of the solvent took place in a culture dish under vacuum for 12 h and then the PLA/TMPTA/nano-ZnO sheet was measured by in situ FTIR, which was heated from 35 °C to 180 °C at a rate of 5 °C·min^−1^, and kept at this temperate for 5 min. The ATR FTIR was applied to analyze the purified PLA samples at room temperature.

### 2.8. Size Exclusion Chromatography (SEC)

The molecular weight (*M_w_*) and molecular weight distribution (MWD) of PLA samples were measured using a size exclusion chromatograph instrument equipped with a triple detection system: a differential refractive index, light scattering, and on-line viscosity measurements of the effluent (model: Waters2414, polystyrene gel columns, Milford, MA, USA). A standard polystyrene sample, PS-105K (*M_w_ ≈* 105,000 g·mol^−1^), was used for calibration. Purified samples were carefully dissolved in chromatographic grade tetrahydrofuran (THF, used as eluting phase) at room temperature for 24 h and filtered, and then injected into the instrument at 35 °C, with a flow rate of 1.0 mL min^−1^. The error margins of the SEC measurements were less than 3%.

### 2.9. Rheological Measurement

#### 2.9.1. Oscillatory Shear Rheology

A rotational rheometer, ARES-G2 (TA Instruments, Newcastle, DE, USA), with a parallel-plate of 25 mm in diameter, was applied to make oscillatory shear rheological measurements with a gap of 1 mm. The dried purified PLA samples, with 0.2 wt % Irganox 1330, were compressed into discs with a thickness of 2.0 mm and diameter of 25 mm at 170 °C under 10 MPa for the measurements. Small amplitude oscillatory shear was performed in the frequency range of 0.1 to 500 rad/s at 170 °C. The applied strain was kept at 1%, so as to ensure that all samples were in the linear viscoelastic regime.

#### 2.9.2. Melt Strength Measurement

The melt extension was performed using a capillary rheometer (Rosand RH7, Malvern, UK) for a Haul-off test at 170 ± 0.5 °C, equipped with a flat entrance angle (180°) of a diameter of 2.0 mm and an aspect ratio of *L*/*D* = 20. The haul-off accelerated speed remained at 5.5 × 10^−4^ m·s^−2^ and the piston speed was kept at 20 mm·min^−1^ throughout the whole testing process.

### 2.10. Differential Scanning Calorimetry (DSC)

The DSC technique was adopted to analyse the thermal properties of pristine PLA and modified PLA samples by using a differential scanning calorimeter, Q10 (TA Instruments, Newcastle, DE, USA). About 10 mg samples sealed in aluminium pans were heated from room temperature to 200 °C at a quick rate of 60 °C·min^−1^, kept for 5 min at this temperature to eliminate the effect of the thermal history, cooled to 40 °C at a rate of 10 °C·min^−1^, and finally reheated to 200 °C at a rate of 10 °C·min^−1^ under nitrogen atmosphere.

### 2.11. Extrusion Foaming Process and Scanning Electron Microscope (SEM)

The PLA granules were extrusion foamed with a twin-screw extruder, (TSSK-3540, whose screw diameter is 35.5 mm and length-diameter ratio is 40:1, Purui Plastics & Rubber Machinery Co. Ltd., Zhangjiagang, China), using azodicarbonamide (AC) as the foaming agent. The temperature of each section was set as 185 °C, 185 °C, 180 °C, 180 °C, 180 °C, 175 °C, 170 °C, 165 °C, 160 °C, and 150 °C, respectively, and the residence time of the material in the process remained 3 ± 0.5 min. Before SEM testing, the foaming specimens were smooth cut in liquid nitrogen atmosphere and treated by desiccation and spray-gold. A scanning electron microscope, KYKY-EM6200 (KYKY Technology, Beijing, China), was applied to analyze the cell structure and morphology of PLA foaming samples. 

## 3. Results and Discussions

### 3.1. Torque Curves and Reaction Evolution

Torque curves for all sample reactions are shown in Figure 1. In order to be observed more clearly, peaks ascribed to the course of material melting are partly omitted and the reaction peaks are emphasized. The increasing torque value, which is proportional to the apparent viscosity (*η_a_*) of materials and dependent on the molecular weight as well as the chain structure of the polymer, indicates the generation of the longer chains or even long branching chains in the reaction system. The curve of pristine PLA displays a monotonous decrease with the mixing time, which is ascribed to the thermal degradation of PLA chains during processing. Z1 and T1 are similar to pristine PLA, but show a lower equilibrium torque value without any new peak, indicating obvious degradation of the PLA chains [36]. However, the torque shows an obviously different trend when the PLA sample reacts with TMPTA facilitated by ZnO nanoparticles. Higher torque values can be obviously found during the whole reaction course compared with that of pristine PLA. Moreover, a new reaction peak appears in all the modified PLA with TMPTA and nano-ZnO indicating a possible competition between degradation and extending or the long chain branching reaction of PLA macromolecules.

### 3.2. ^1^H-NMR Spectrum

**Figure 2:**
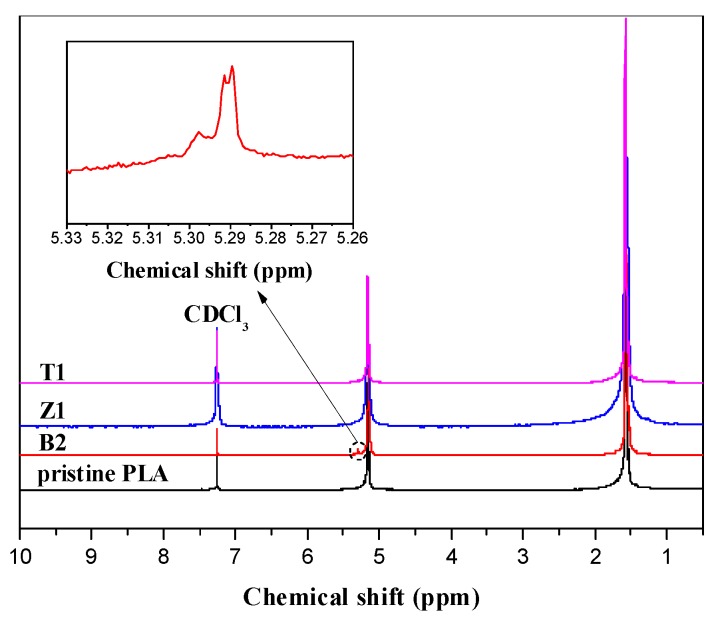
^1^H-NMR spectra of PLA samples.

The structures of the purified PLA samples are investigated in detail using the ^1^H-NMR spectrum. The typical spectra of PLA samples are presented in Figure 2. For pristine PLA, the typical resonances at 1.57 and 5.15 ppm, respectively, correspond to the methyl (–CH_3_) and tertiary hydrogen (–CH) on the PLA backbone. For the modified Sample B2, an apparent new resonance at about 5.30 ppm can be observed, which is due to the hydrogen atoms of the double bond (–CH=CH_2_) in the monomer TMPTA, indicating that the acrylate monomer was grafted onto the PLA backbone and the double bond (C=C) was not reacted with the PLA chains. As a result, the monomer TMPTA grafted on PLA can almost come from the transesterification reaction facilitated by nano-ZnO. However, this double bond resonance is absent in both Z1 and T1, indicating that the grafting reaction cannot take place only depending on nano-ZnO or TMPTA alone. Given the tri-functionality of the ester group in TMPTA, some kind of three-arm star-like branched PLA chains may be formed. The relative grafting percentages (RGP) of the monomer could be calculated according to RGP = IA_CH=CH_/IA_total_, where IA_CH=CH_ and IA_total_ are the integral areas of double bond hydrogen atom resonance and the total intensity of hydrogen atoms, respectively. The RGP values are summarized in Table 1. For the samples modified by TMPTA and nano-ZnO, the RGP varies with the increasing dosage of nano-ZnO, and when 0.4 phr nano-ZnO is added in this system, Sample B2 exhibits a relatively high RGP value, indicating that the dosage of the accelerants has a very important role in the occurrence of branching reactions.

### 3.3. X-ray Photoelectron Spectroscopy (XPS)

**Figure 3:**
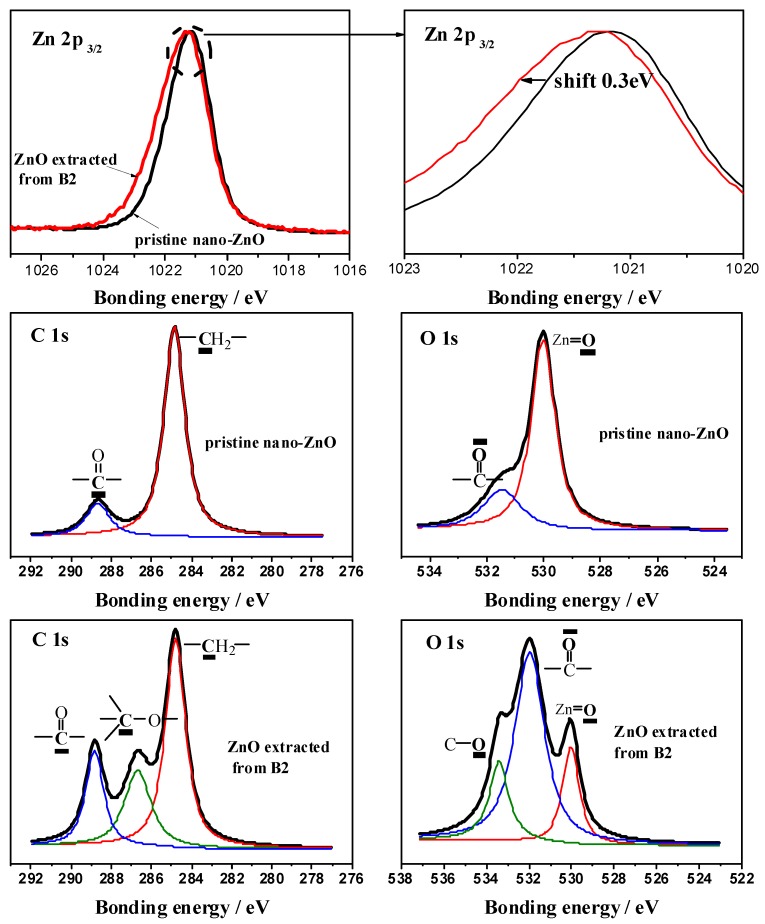
Zn 2p_3/2_, C 1s, and O 1s high-resolution XPS spectra recorded at a 90° takeoff angle for ZnO at room temperature.

High-resolution scans of the Zn 2p_3/2_, C 1s, and O 1s photoelectron regions of the pristine nano-ZnO and extracted ZnO from B2 are respectively shown in Figure 3. One peak at 1021.7 eV corresponding to Zn 2p_3/2_ for Zn=O bonds in pristine nano-ZnO can be observed, while there is about a 0.3 eV shift towards a high bonding energy for the peak of ZnO extracted from B2, indicating that the coordination effect between the Zn atom and carbonyl oxygen in the ester group decreases the electron cloud density of Zn atoms. Curves of C 1s and O 1s are fitted with multiple Gaussians with a Lorentzian contribution of 10%. In pristine nano-ZnO, there are two carbon types that correspond to the C–H and the carbonyl, and two oxygen types that correspond to the lattice oxygens and carbonyl on its surface. For ZnO extracted from B2, a stronger peak for the double bond of carbon to oxygen (C=O) and a new response corresponding to carbon-oxygen bonds (C–O) can be observed compared with the pristine nano-ZnO which belongs to the ester group in PLA, revealing the gathering of PLA chains and the coordination effect with nano-ZnO on the surface. The intensity augment of C=O and the appearance of the C–O peak can be ascribed to the residual PLA which cannot be extracted into dichloromethane due to the complexation reaction.

### 3.4. FTIR Spectroscopy

**Figure 4:**
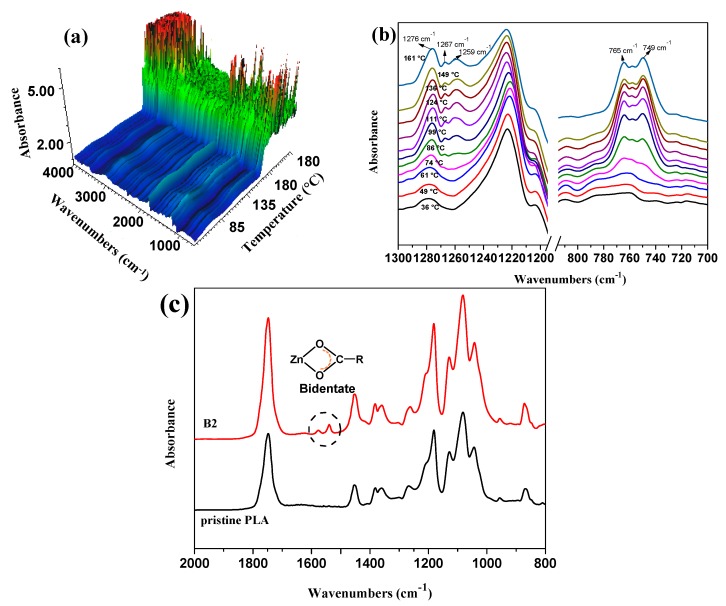
(**a**) 3D image of in situ FTIR of PLA/TMPTA/nano-ZnO blend; (**b**) in situ FTIR bands in the range of 1300–700 cm^−1^ at different temperatures before melting; and (**c**) ATR FTIR curves of purified Sample B2 and pristine PLA.

FTIR results of PLA samples are shown in Figure 4. As shown in Figure 4a,b, some variations can be observed for the PLA/TMPTA/nano-ZnO blend with the rise of temperature. Bands at 1276 cm^−1^ for the C–O vibration and bands at 762 cm^−1^ for C=O can be found in the blend owing to the ester groups in PLA and TMPTA, which increase with the rise of temperature. In addition, extra peaks appear at 1259 cm^−1^ and 1267 cm^−1^ when the temperature exceeds 86 °C, which are also observed in the zinc lactate with Zn being the coordination centre, indicating the coordination reaction between C-O bonds and Zn atoms. The peak for C=O at 765 cm^−1^ is divided gradually with the increasing temperature, which is perhaps due to the conjugative effect that comes from the C=C and C=O conjugated double bonds introduced by TMPTA and as a result, the peak shifts to the low wavenumber region. However, peaks become so strong after the melting of PLA when the temperature has reached 180 °C because of the augment in the exposed characteristic groups. Figure 4c shows the ATR FTIR curves of purified Sample B2 and pristine PLA. It can be found that obvious additional characteristic peaks for zinc carboxylate appear at 1578 cm^−1^ and 1539 cm^−1^ compared with pristine PLA, which further verifies the coordination reaction and the bidentate form can be ascertained by the wavenumber difference value between *v*_C–O_ (1451 cm^−1^) and *v*_C=O_ (1540 cm^−1^) [37].

**Figure 5:**
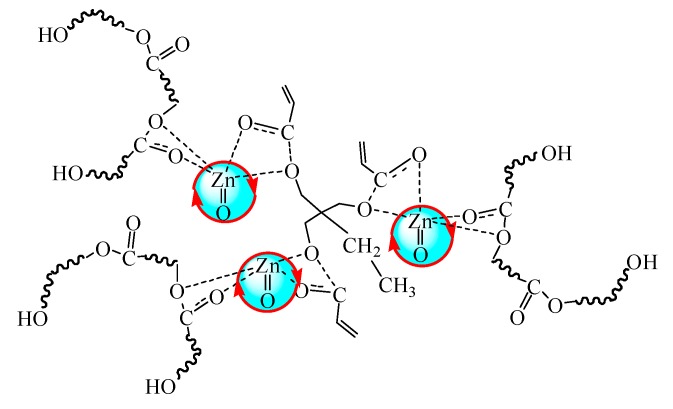
Schematic diagram of the coordination effect of nano-ZnO with PLA chains and the monomer TMPTA.

The coordination effect of nano-ZnO with PLA chains and the monomer TMPTA is illustrated in Figure 5. Macromolecular PLA chains gather around the surface of nano-ZnO benefiting from the hydrophilic groups at the end of the PLA chains, and the electron cloud further moves to the carbonyl oxygen owing to the complexation between nano-ZnO and ester carbonyl in PLA and TMPTA, which decreases the bonding energy of C–O in the ester group and activates the carbon-oxygen bond [38]. The C–O bond tends to break and then the adjacent micromolecular tri-functional monomer TMPTA combines with PLA long chains by an ester exchange reaction. As a result, a kind of three-arm star-shaped LCBPLA can be obtained.

### 3.5. Proposed Mechanism

**Figure 6:**
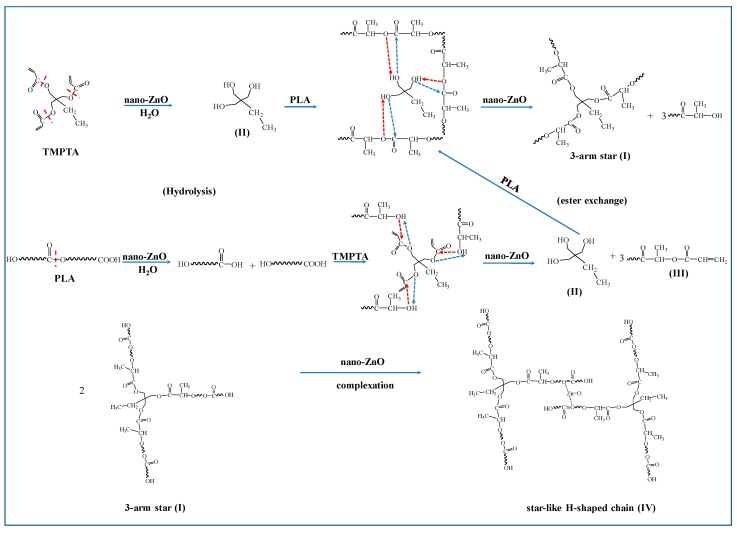
The proposed reaction mechanism for the exchange reaction of PLA with TMPTA accelerated by nano-ZnO.

The results of the reaction torque curve, ^1^H-NMR, XPS, and FTIR have shown above that ester exchange between PLA and TMPTA can take place in the presence of nano-ZnO. The proposed branching reaction mechanism of PLA is inferred in Figure 6, as well as the corresponding most possible topological chain structure existing in the system. Firstly, PLA end-capped with hydroxy (or carboxyl) and trimethylolpropane (TMP, II) can be achieved, which respectively come from the hydrolysis of the ester groups in the main chains of PLA and the monomer TMPTA under the action of environmental water and nano-ZnO in this system. Following this, an ester exchange reaction between hydroxyls (or carboxyls) and ester groups can take place with the acceleration of nano-ZnO [39]. Especially for the transesterification between TMP and long PLA chains, some kind of three-arm star-like PLA chains (I) can be obtained owing to the ester exchange reaction and at the same time, unsaturated polyesters (III) will be generated as coproducts. Under the action of nano-ZnO, two three-arm star PLA chains (I) gather around the ZnO nanoparticles and coordinate with them via the complexation between Zn atoms in nano-ZnO and O atoms in PLA, thus the two three-arm star PLA chains (I) are connected. As a result, some kind of star-like H-shaped PLA chains (IV) can be obtained.

### 3.6. Size Exclusion Chromatography (SEC)

**Figure 7:**
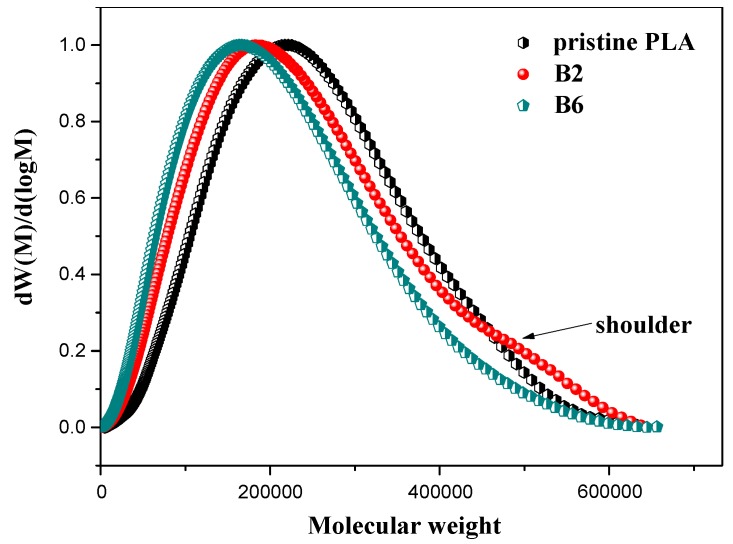
Molecular weight distributions of pristine PLA and modified samples.

The molecular weight distribution (MWD) of PLA samples is shown in Figure 7, and the corresponding data are listed in Table 2. As shown, the curves of Sample B2 and B6 get broader and shift to a lower *M_w_* region compared with the pristine PLA, indicating that the degradation reaction took place during the melt transesterification process. An evident shoulder can be seen at the high molecular weight region on the curve of B2, which corresponded to the sparsely long branching components [40,41]. However, the shoulder disappears in the curve of B6, whose melt blending time was up to 400 s, indicating that obvious chain degradation occurs owing to the long-time melt transesterification reaction and more and more short chains are generated, which is also reflected in the molecular weight value.

### 3.7. Rheological Measurements

**Figure 8:**
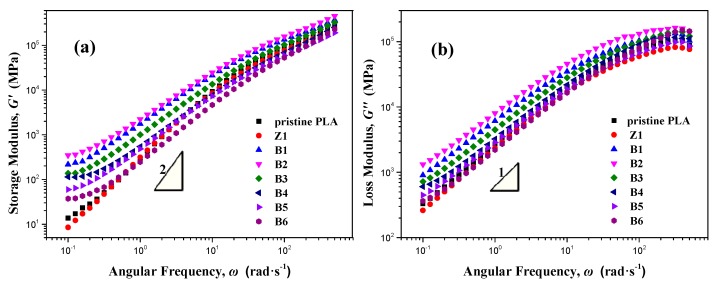
Rheological plots of PLA samples at 170 °C obtained from frequency sweeps: (**a**) storage modulus *G*′ and (**b**) loss modulus *G*″ as a function of angular frequency.

Rheological properties of polymers in the linear viscoelastic region are very sensitive to molecule topological structure changes. Some distinct rheological behaviors of polymers in the presence of LCB were described [42]. Complex shear moduli obtained from frequency sweeps of pristine PLA and modified samples at 170 °C are shown in Figure 8. The pristine PLA and Sample Z1 exhibit the typical terminal behavior which conforms to the well-known frequency dependence for a linear polymer, i.e., *G*′∝*ω*^2^ and *G*″∝*ω* [43], indicating their linear chain structure. The samples modified by TMPTA and nano-ZnO deviate from the terminal behavior. For B1, B2, B3, B4, and B5, the enhancement in *G*″ is limited, while a great increase in the storage modulus at a low frequency could be obviously observed compared with pristine PLA, indicating a longer relaxation mechanism which could be ascribed to the formation of branched chains. For B6, sampled from the reaction end, obvious decreases in both *G*′ and *G*″ are presented, which can be attributed to the degradation of the PLA chains during the long mixing process in the presence of nano-ZnO. Those above are also reflected in the melt flow rate (MFR) and zero shear viscosity (*η*_0_) listed in Table 1: the higher *G*′ corresponds to lower MFR and higher *η*_0_.

**Figure 9:**
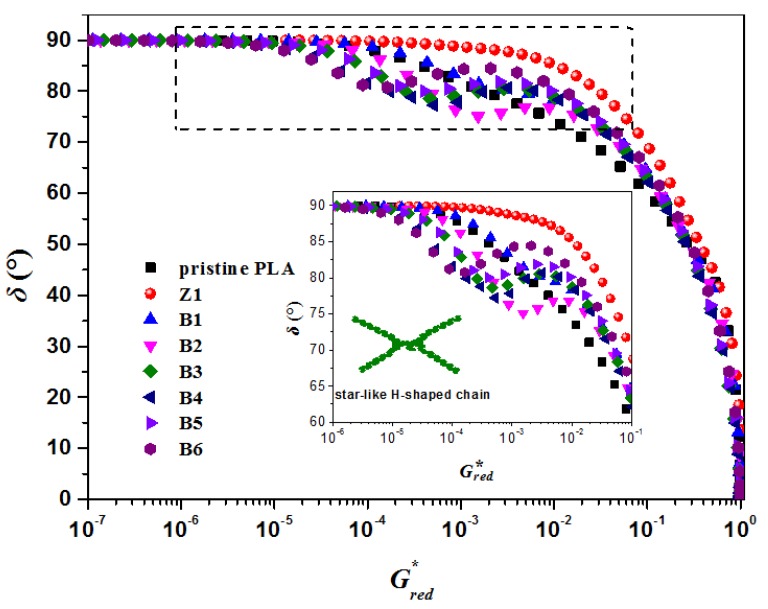
Reduced vGP plots for pristine PLA and modified samples at 170 °C.

The chain topology of polymers can be illustrated by a vGP plot, firstly proposed by van Gurp and Palme [44]. Plotting phase angle (*δ*) vs. complex modulus (|G*|) was found to be very sensitive to the topological structure type of LCB in the polymer chains. In order to eliminate the effect of differences among the molecular weight, molecular weight distribution, and relaxation time of PLA samples, the reduced vGP plots are applied and shown in Figure 9 ($G_{red}^{*}$ = |G*|/$G_{N}^{0}$, where $G_{N}^{0}$ is the plateau modulus). As shown, plots of pristine PLA and Sample Z1 exhibit a typical shape for a linear polymer, whose phase angle decreases monotonically with the complex modulus without any kinds of transitions when |G*| is smaller than $G_{N}^{0}$. An evident inflection point and sharp bump appear in the curves of other modified samples, indicating a longer relaxation process and the existence of LCBs. It was reported that the position of the inflection point and the shape of vGP plots could definitely reflect the chain type of the LCB polymers [45] due to the different relaxation for LCB polymers with different chain structures. For these modified samples, the vGP plots present the shape of a “bump”, and the inflection points appear at a high *δ* value (about 75–80°) and a relatively low $G_{red}^{*}$ value, which accords with the characteristic of a star-like H-shaped polymer, indicating that the topological structure of the modified samples is star-like H-shaped. These star-like H-shaped chains are obtained from the double or multiple ester exchange of the PLA arms and TMPTA, which possesses an H-shaped topological structure with a negligible short joining chain between two long arms and that is star-like from an overall view. Moreover, it was reported that the vGP plot shifts to the position of a lower phase angle with the increasing level of LCB [45,46,47]. The lower the phase angle is, the stronger the entanglement becomes. Therefore, it can be observed that B2 possesses the highest LCB level and B5 owns relatively less LCB. In addition, compared with Sample B2, the inflection point of B6 appears at a higher phase angle, indicating the decrease in LCB level, which can be ascribed to the degradation of PLA chains and the formation of lots of short branches during the long mixing process.

The tube model used to estimate the degree of branching chains (*X*) is applied here to further confirm the star shape of the PLA chains [48,49,50]. The branching degree (*X*) can be described by Equation (1) as follows:

(1) $$X\approx \frac{ln\left(\frac{\eta_{BL}}{\eta_{L}}\right)}{\upsilon \left[\left(\frac{M_{L}}{M_{C}}\right)-1\right]-3ln\left(\frac{M_{L}}{M_{C}}\right)}\;$$

Here, *υ* is a structure constant of polymers which is related to the structure of polymer chains and is independent on the number of branching chains. For LCB polymers, *υ* can be used to ascertain the LCB type [50,51,52]. *η*_BL_ and *η*_L_ are the zero-shear viscosity of branching chains and the linear chains, respectively, which can be obtained from the rheological measurements. *M*_L_ is the molecular weight of linear PLA and *M*_C_ is the molecular weight at the onset of entanglements, a material constant that depends on molecular rigidity and equals twice the molecular weight between two successive entanglements: *M*_C_ ≈ 2*M*_e_. For PLA, *M*_e_ is roughly equal to 8300 [53]. The molecular weight of linear pristine PLA can be expressed with the melt viscosity at the entanglement crossover (*η*_C_) as Equation (2):

(2) $$M_{L}=M_{C}{\left(\frac{\eta_{L}}{\eta_{C}}\right)}^{1/3.5}\;$$

Thus, the critical viscosity *η*_C_ can be calculated. Then, Equation (1) can be restated as follows:

(3) $$X\approx \frac{ln\left(\frac{\eta_{BL}}{\eta_{L}}\right)}{\upsilon \left[{\left(\frac{\eta_{L}}{\eta_{C}}\right)}^{1/3.5}-1\right]-\frac{6}{7}ln\left(\frac{\eta_{L}}{\eta_{C}}\right)}\;$$

The branching frequency (*X*) values can be acquired by a branching calculation module in an SEC instrument with a triple detector. Thus, all parameters except for *υ* in Formula (3) are acquired, and the structure constant *υ* can be calculated.

**Figure 10:**
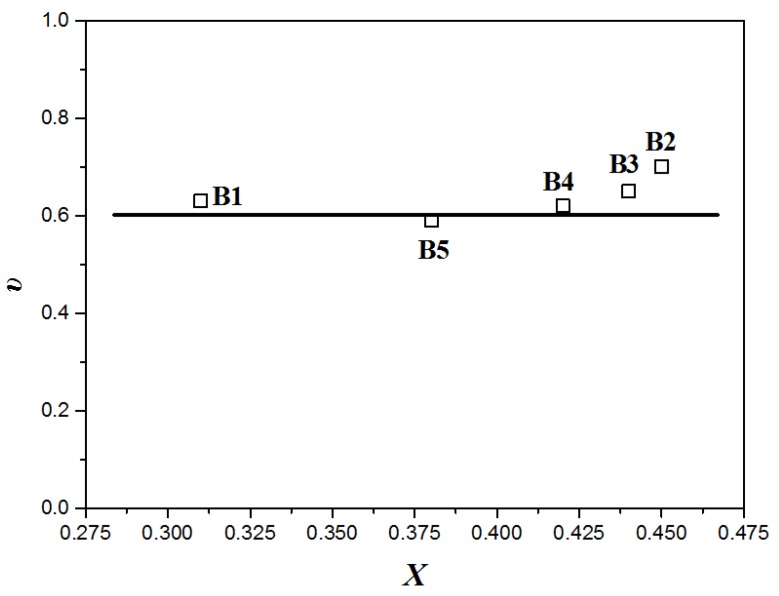
The relationship of structure constant (*υ*) with branching frequency (*X*).

As shown in Figure 10, the values of the structure constant *υ* for all the modified samples are in the neighborhood of 0.6, which is consistent with previous reports for star-shaped polymers published by Pearson, certainly confirming that the topological structure of LCBPLA prepared in our study is star-shaped (for the strict H-shaped polymers, *υ* = 1.2) [49,50,51].

**Figure 11:**
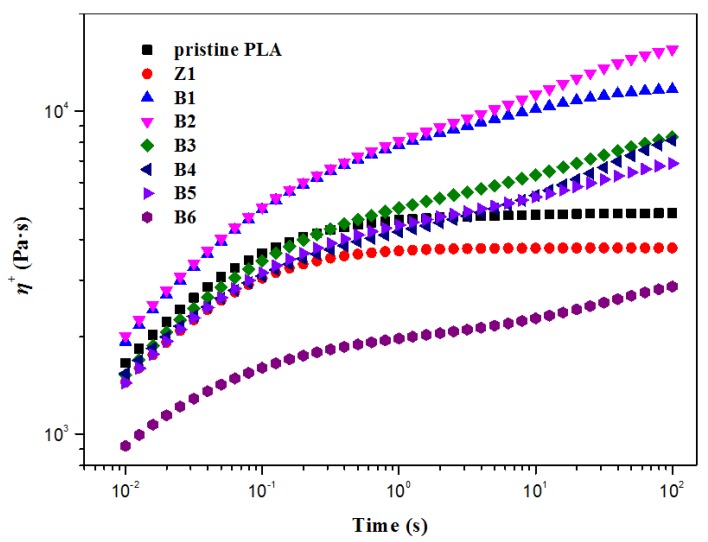
Tensile stress growth coefficients η^+^ of PLA samples at a strain rate of 1.0 s^−1^.

An extensional flow experiment is usually applied to characterize the extensional behavior of polymers. When LCB exists, PLA melt can obviously exhibit strain hardening behavior. It was known from the literature that the extensional flow data can be fitted using parameters determined solely by the shear measurements [54,55]. Figure 11 shows the tensile stress growth coefficients *η*^+^ of the melt of pristine PLA and modified PLA samples at a strain rate of 1.0 s^−1^. As shown, *η*^+^ first increases with the run time and then levels off without evident strain hardening for pristine PLA and Sample Z1; in contrast, the samples modified by TMPTA and nano-ZnO display obvious strain hardening behavior. Sample B2 presents high and increasing *η*^+^ along with the run time in the whole range of the measurement, indicating fairly prominent strain hardening behavior, which is attributed to the star-like LCB enhancing the entanglement among the PLA chains.

**Figure 12:**
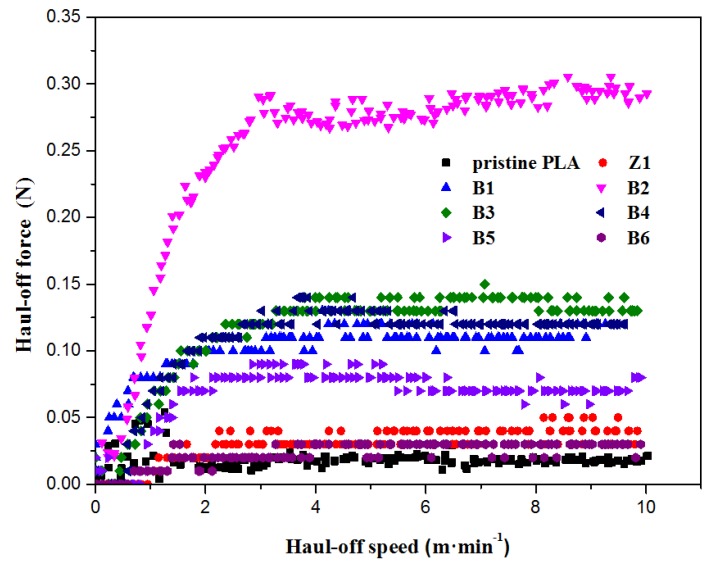
Haul-off force vs. Haul-off speed of PLA samples at 170 °C.

Another direct characteristic of long branching chains in polymers is the improvement in melt strength. When LCB exists, a polymer often exhibits a high melt strength. Figure 12 shows the Haul-off test curves for PLA samples and the “melt strength” is defined as the maximum force before the melt strand breaking. For pristine PLA, the melt strength is just about 0.01 N, while that of the modified samples (B1–B5) is much higher than that value, especially for B2, which has reached to more than 0.30 N, indicating that the introduction of the star-like LCBs can greatly improve the melt strength of PLA. However, B6 displays a relatively low melt strength because of the excessive transesterification reactions and degradation of PLA chains during the long-time mixing process. In addition, the rheological properties of the Sample Z1 with nano-ZnO alone are similar to the linear polymer, indicating that nano-ZnO cannot change the topological structure of PLA without the monomer TMPTA.

### 3.8. SEM Images of Foaming PLA

**Figure 13:**
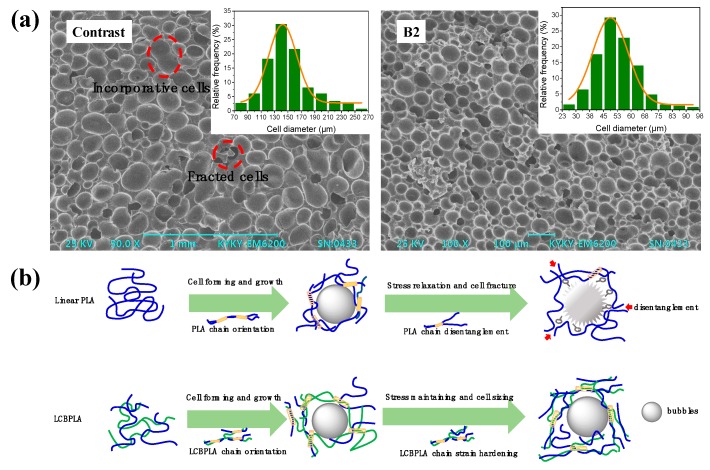
(**a**) SEM images and cell diameter distribution of extrusion foaming PLA; (**b**) Extrusion foaming behavior of linear PLA and LCBPLA melts.

Enhancement in the melt strength of polymers can obviously improve their foaming behaviors. Figure 13 shows the SEM photos and cell diameter distribution of extrusion foaming PLA samples. In order to eliminate the influence of nano-ZnO on the nucleation of cells, pristine PLA with 0.4 phr nano-ZnO was extrusion foamed as a comparison. As shown, incorporative cells and fracted cells can be observed in the contrastive sample due to its poor melt strength and the mean cell diameter has reached 140 μm. While by comparison, foamed Sample B2 displays a much smaller mean cell diameter (52.13 μm), higher cell density, and less fracted cells, which benefits from the improvement of melt strength and strain hardening owing to the introduction of LCB structures in PLA. Bubbles grow quickly and the cell diameter increases rapidly under the quick pressure relief at the primary stage of cell formation [56]. In this stage, the cell wall suffers quick stretching and the prominent elastic response of LCBPLA melt can restrain the cell growth, which decreases the cell diameter. Then, the cells grow gradually and the accumulated stress relaxes, which reduces the strength of the cell wall and incorporative cells can be formed. For Sample B2, highly entangled long branching chains are capable of accumulating more stresses and restrain the relaxation, and as a result, smaller separated cells can be seized and obtained in the final foams.

### 3.9. Crystallization Behavior

**Figure 14:**
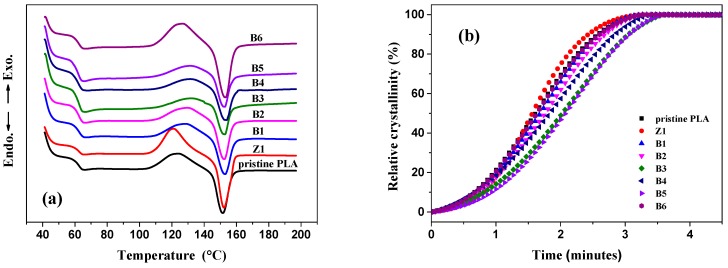
(**a**) DSC second heating curves and (**b**) relative cold crystallinity versus time of PLA samples at a heating rate of 10 °C·min^−1^.

Figure 14a shows the second heating curves of pristine PLA and modified samples at the scanning rate of 10 °C·min^−1^ after eliminating the thermal history at 200 °C for 5 min, and detailed data are listed in Table 3. The glass transition (*T*_g_) and melting (*T*_m_) temperatures of all linear and LCB-PLAs are very similar at almost 62.6 °C and 152 °C, respectively. An exothermal peak related to the cold crystallization behavior of PLA samples appears after the glass transition. Converting temperature (counted from *T*_onset_) to time via the heating rate, the relative cold crystallinity versus time can be shown in Figure 14b and the crystallization rate, *v*, can be calculated by *v* = 1/*t*_1/2_, where *t*_1/2_ is the time when the relative crystallinity reaches 50%. As shown, Sample Z1 displays a little earlier onset cold crystallization temperature (*T*_onset_) and crystallization peak temperature (*T*_cp_) than those of pristine PLA owing to the heterogeneous nucleation of nano-ZnO, which is also shown in its higher difference value ∆*H*_m_ − ∆*H*_c_, and moreover, a faster crystallization rate can also be observed. On the contrary, *T*_onset_ and *T*_cp_ values of LCB samples modified by TMPTA and nano-ZnO (B1–B5) shift to a higher temperature, and a lower crystallization rate as well as melting enthalpies (∆*H*_m_) can be observed, which is different from the LCBPLA prepared by a radical reaction [57,58,59]. The most likely explanation for this is that the highly long branching structures in PLA chains are more difficult to crystallize than pristine linear PLA, which is generated by a proper transesterification reaction manifested as a narrow reaction peak (about 30 s) in the torque curve (see Figure 1). In addition, nano-ZnO in this LCB system can collect the long-branched chains produced by the transesterification reaction between PLA macromolecules and TMPTA with the adsorption on its surface, thus restricting the movement of long PLA branches and decreasing their ordering ability. These limited branches cannot crystallize until they can break away from the bondage at a higher temperature. As shown, the values of *T*_onset_ and *T*_cp_ both increase with the increasing dosage of nano-ZnO, indicating a stronger binding effect from the increasing number of nano-ZnOs. However, B6 shows a relatively high melting enthalpy value, which can be attributed to the vast short linear molecules generated by the degradation of LCBPLA chains during the long-time mixing.

## 4. Conclusions

In this study, a kind of long chain branched PLA was prepared via the transesterification between PLA chains and the tri-functional monomer TMPTA during melt blending facilitated by nano-ZnO in a torque rheometer, and the LCB samples were obtained at the optimal reaction time corresponding to the top of the reactive peak in the torque curves. ^1^H-NMR, XPS, and FTIR results showed that PLA long chains reacted with the monomer TMPTA via transesterification accelerated by nano-ZnO owing to the coordination effect of nano-ZnO with PLA chains and the monomer TMPTA. GPC and rheological measurements revealed that sparse LCB structures were formed in the moderate melt branching process with the addition of a tri-functional monomer and nano-ZnO. VGP plots as well as the structure constant *υ* further confirmed that the topological structure of the LCBPLA was star-like shaped. It was found that the nano-ZnO content and sampling occasion had very important roles in the occurrence of branching reactions and the sparse LCB structure could affect the melt strength as well as the extrusion foaming behavior of PLA products. When the dosage of nano-ZnO was 0.4 phr, the Sample B2 sampled at the reaction peak in the torque curve exhibited obvious strain hardening behavior in the melt state and the melt strength reached over 0.30 N, which apparently decreased the cell diameter and increased the cell density of the extrusion foaming specimen. However, severe degradation took place under a mixing time that was too long and lots of short linear chains were generated due to the excessive transesterification reaction, which resulted in a sharp decline in melt strength. The cold crystallization was found to be harder due to the introduction of the star-like LCBs and nano-ZnO in this system, which reduce the regularity of PLA chains and restrict the movement of long PLA branches. Nano-ZnO and the LCB structures in the system had synergistic effects on the crystallization behavior of the LCBPLA.

## Figures and Tables

**Table 1:** Formulation of the experiment and the corresponding measurement data.

Sample	PLA Pellet (g)	Nano-ZnO (g)	TMPTA (g)	Sampling Time (s) ^a^	GP (%)	MFR ^b^ (g/10 min)	*η*_0_ (Pa·s) ^c^
PLA	100	0	0	400	0	4.10	4571.41
Z1	100	0.40	0	400	0	4.70	4387.42
T1	100	0	2.00	400	0	4.52	4408.62
B1	100	0.20	2.00	161 *	0.29	1.08	11,206.2
B2	100	0.40	2.00	145 *	0.38	0.98	11,963.8
B3	100	0.60	2.00	150 *	0.33	1.54	8179.90
B4	100	0.80	2.00	145 *	0.35	2.44	5955.33
B5	100	1.00	2.00	155 *	0.26	2.82	6672.38
B6	100	0.40	2.00	400	0.36	5.92	2176.05

**^a^** The superscript “*” refers to the specimens that were sampled at the “reaction peak” according to the mixing curve shown in Figure 1. **^b^** Measured at 190 °C and 2.16 kg. **^c^** *η*_0_ values are approximated by the η* values extrapolated to *ω* = 0.01 rad·s^−1^.

**Table 2:** Molecular weight and its distribution of samples.

Sample	*M_n_* (g/mol)	*M_w_* (g/mol)	*M_w_*/*M_n_*
PLA	108,994	185,293	1.70
B2	97,055	178,689	1.84
B6	74,277	126,084	1.82

**Table 3:** Second heating crystallization parameters of samples at a 10 °C·min^−1^ heating rate.

Sample	*T*_g_ (°C)	*T*_onset_ (°C)	*T*_cp_ (°C)	*T*_m_ (°C)	∆*H*_m_ (J/g)	∆*H*_m_ − ∆*H*_c_ (J/g)	*v* × 10^−3^ (s^−1^)
PLA	62.60	106.99	124.10	151.54	15.520	1.260	10.30
Z1	62.61	105.60	120.84	152.32	18.731	2.424	10.60
B1	62.79	108.17	128.49	152.79	11.600	1.899	9.96
B2	62.63	109.58	129.67	152.47	8.710	0.544	9.57
B3	62.68	110.98	131.94	152.90	4.394	0.499	8.17
B4	62.67	112.83	131.01	153.45	6.803	0.903	9.16
B5	62.63	111.00	131.21	152.68	5.053	0.421	8.05
B6	62.64	109.76	126.51	153.04	14.31	0.640	10.20
